# Software patterns and data structures for the runtime coordination of robots, with a focus on real-time execution performance

**DOI:** 10.3389/frobt.2024.1363041

**Published:** 2024-09-04

**Authors:** Maria I. Artigas, Rômulo T. Rodrigues, Lars Vanderseypen, Herman Bruyninckx

**Affiliations:** ^1^ Department of Mechanical Engineering, KU Leuven, Leuven, Belgium; ^2^ Flanders Make, Leuven, Belgium; ^3^ Department of Mechanical Engineering, TU Eindhoven, Eindhoven, Netherlands

**Keywords:** multi-robot, coordination, Petri net, finite state machine, real-time, shared memory

## Abstract

This paper introduces software patterns (registration, acquire-release, and cache awareness) and data structures (Petri net, finite state machine, and protocol flag array) to support the coordinated execution of software activities (also called “components” or “agents”). Moreover, it presents and tests an implementation for Petri nets that supports real-time execution in shared memory for deployment inside one individual robot and separates event firing and handling, enabling distributed deployment between multiple robots. Experimental validation of the introduced patterns and data structures is performed within the context of activities for task execution, control and perception, and decision making for an application on coordinated navigation.

## 1 Introduction

Society expects “smarter” robotics technology and “higher performance” of the applications and systems that are built with it. A major contribution toward realizing these expectations is improving the capabilities and the predictability of the composition of robotic components into systems. Coordination plays a major role in achieving this predictability: a system has several concurrently active components that require access to “resources” that cannot be shared trivially, such as locations in space or tools and sensors. Application developers must translate user requirements into concrete *coordination specifications*: when and why each of the components in the system must start or stop a particular “behavior.” Coordination is triggered by “events” generated by the software component in the system that has the authority to make such decisions, and it is provided with the necessary information by all the components that rely on its coordination. A good (but not necessarily unique) *separation of concerns* (Dijkstra, 1982) approach to ensure coordinated resource sharing with predictable performance and acceptable access policies is to introduce a *dedicated coordination software component* for each shared resource. The contributions of this paper are focused on this coordination design concern.

**FIGURE 1:**
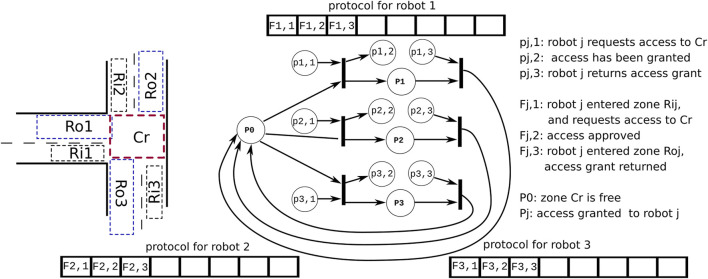
Coordination between concurrently active “agents” in traffic situations, particularly a T-junction. Left: only one “robot” coming from one of the three roads shall be allowed to access the crossing Cr. Right: our design introduces a *mediator* software component to realize such coordination problems. It relies on i) a *Petri net* as a *declarative model* of the coordination’s decision making and ii) a *protocol* between the mediator and each of the coordinated robots, via which the latter’s own internal decision making is *decoupled* from that of all other robots.

The left-hand side of Figure 1 shows a simple example of the role of coordination in multi-robot systems (Section 1.3 provides an overview of more archetypical coordination-use cases).

• The sketch on the left-hand side represents a “T junction.”

• Robots can come from three different roads, each with the timing unknown to the other robots.

• The “crossing area” Cr is the “shared resource” that should be entered by only one robot at a time.

The figure’s right-hand side sketches our software design (which is described in detail in the later sections of this paper).

• The crossing area gets its own *mediator* software component (Gamma et al., 1995). The mediator allows robots to navigate the crossing area in a coordinated way. The core data structure of the mediator is a *Petri net* that represents a *declarative model* of the coordination’s decision-making.

• The second software component is a *map* data structure that all robots share with the mediator. On that map, they indicate which area they are currently driving in. These areas are given numbers 1, 2, and 3 for each of the three roads “R”; “i” and “o” indicate the “incoming” and “outgoing” lanes.

• The third software component is a *protocol* data structure that is accessed in sequence by the mediator and each of the coordinated robots. The protocol decouples a robot’s own internal decision making from that of all other robots.

The *map* is also a shared resource in itself, but its software design presents a different set of coordination challenges, which are beyond the scope of this paper; for further details, refer to Van Baelen et al. (2022).

The following sub-sections introduce and define all the concepts needed in this paper. Section 2 discusses the previous work on which this paper is based and other related work. Section 3 describes the coordination mechanisms introduced in this paper and the complementary communication and configuration mechanisms for its integration. Section 4 introduces the implementation and evaluation of the Petri nets for runtime coordination. Section 5 explains the application of the previously described patterns in a coordinated navigation case. A secondary demonstration is also provided. Section 6 concludes the paper with a discussion of the presented and future work. Supplementary Appendix SA explains the connection between the coordinating and coordinated activities via events.

### 1.1 Component

The terminology “(software) components” has been interpreted several times over the past decades (Brugali and Scandurra, 2009; Brugali and Shakhimardanov, 2010), referring to the software primitive that provides “computational behavior” to a system. The terminology used in this paper to represent complementary types of computational behavior is as follows:

• (robot) Component: each piece of software-controlled hardware that the application identifies as a “robot.” It is not to be subdivided further hardware-wise and can be connected to other robot components via mechanical, power, and information connectors to form a larger “composite” robot component.

• Computer: the set of CPUs, each with possibly several computing cores and managed by one operating system. Many robot *components* are built with more than one computer.

• Process and thread: the two well-known *application-independent* computational primitives under the control of an operating system.

• Activity: the smallest concurrently running piece of software that components the need and is deployed in a thread. Typically, each component requires application-centric functionalities implemented in a multitude of activities, all running *asynchronously* on the same computer or different computers.

• Algorithm: an activity can execute one or more algorithms inside, for which it guarantees the *synchronous* execution context needed to realize the *functionalities* (or “*behavior*”) of a component. In other words, the activity is responsible for asynchronous data exchange between activities, making sure that their algorithms only have to access locally stored data structures that are, hence, synchronously processed.

One could have given the name software component to what is called *activity* above. Because activities are designed to be executed concurrently, an appropriate set of asynchronous data exchange mechanisms is needed; these mechanisms should be shared between the activities within the same process memory or use one or more inter-process communication technologies. The challenges of data consistency between concurrently running activities are to be solved at the activity level but not at the thread or algorithm levels. The thread level in a software component design is responsible for scheduling by the operating system. The process level is responsible for managing resources shared between all activities within all process’s threads, such as file descriptors, signal handling, and thread priorities.

### 1.2 Coordination

Coordination is all decision making shared between concurrently executing *activities* about which of their *algorithms* (“behaviors”) must become “(in)active” at each moment in time in each of the robot components and about how to keep other robot components informed about which behavior(s) are currently “(in)active.” A key message of this paper is that all forms of inter-activity coordination can be realized with the following primitives, whose “separation of concerns” roles (Dijkstra, 1982) are illustrated in Figure 1.

• Flag: this represents the “state” of a *Boolean condition* defined over a set of parameters in the behavior(s) of an activity. For example, for mobile robots navigating in the neighborhood of the crossing in Figure 1, flags can indicate areas in which each mobile robot finds itself. (The above-mentioned “map” software component could act as the major source of *flag* information and *event* information introduced below.)

• Event: this represents the change in the Boolean state of a flag. Because Figure 1 is a static “snapshot” of the status of the world, it does not show *events.* They only come in when the time-varying *dynamics* of the coordination problem are considered and they are to be *communicated* between activities.

• Finite state machine [FSM, Hrúz and Zhou (2007)]: each of the activities needs to realize a particular behavior in a particular order. Such an order is represented *declaratively* by a finite state machine data structure and behavior:

• Each activity can be “in” *one and only one state* at a time.

• In each state of the finite state machine, the activity executes a particular set of algorithms and communicates a particular set of data structures, including *events.*

• Transitions between states are triggered by incoming events or events generated internally in the activity.

• Some of these transitions can also give rise to the *firing* of events that must be communicated to other activities.

This description of the *mechanism* of an FSM corresponds to that of a *Mealy machine* (Mealy, 1955), which is formally represented as a tuple (S,I,O,T,O) , with S representing the finite set of *states*; I representing the finite set of *input* events (or “input symbols”); and O representing the finite set of *output* events (or “output symbols”).

• T : the *transition function* T:S×I→S maps the combination of a *state* and an *input* event to a *state.*

• O : the output function O:S×I→O maps the combination of a state and an input event to an output event.

In the actual execution of an FSM, the *policy* must be added to select one of the states as the *initial state* $S_{0}$ .

• Petri net (PN): this is a data structure that keeps track of the (externally exposed) state of a set of activities that need to be coordinated in the coordination *mediator* software component, as shown in Figure 1. Each of these states fills a *place* in the Petri net with a *token.* (This paper uses only the simplest form of Petri nets, sometimes called *safe* Petri nets (Barylska et al., 2017), in which each place can hold only zero or one *token.*) The role of the Petri net is to support decisions about the coordination *between activities* and not about the *internal algorithm* coordination of one single activity. *Semantically*, a Petri net can have more than one of its places *marked* at any given time, while a finite state machine can *be* in only one of its states at any given time.

This *mechanism*of a Petri net is formally represented as a tuple (P,T,M,F) , with P representing the finite set of *places*; T representing the finite set of *transitions*( P and T are always disjoint); M representing the set of *markings*of the Petri net, where each marking is a mapping M:P×{0,1} , indicating whether a *place*is marked or not, that is, it contains a *token*or not; and F representing the *firing function*such that F:P×M→M removes the *tokens*in the input *places*of a *transition*whenever all these *places*are *marked*and produces a *token*in each of the *transition’*s output *places.*

In the actual execution of a Petri net, the *policy* must be added to define an *initial marking* $M_{0}$ .

• Protocol: this represents the order in which a particular subset of flags is allowed to be set to “true” in the interaction between the coordination *mediator* and *one* of the coordinated activities. Such an order must be agreed upon *in advance* by all activities participating in the coordination mediation to be able to guarantee temporal constraints between behavior state changes.

For example, the protocols in Figure 1 show that for each robot, the sequence of execution is as follows: 1) the robot requests access, 2) the access is approved, and 3) the robot can enter the area.

Note that the “array” used in Figure 1 to represent a protocol is *always finite*, and flag entries are entered always from the first entry on the left. In other words, it is *not* an endlessly growing “stream” of flag entries. When the protocol ends, for one reason or another, all entries are removed so that the next execution of the protocol starts with an empty array again.

In the simple *workspace sharing* example in Figure 1, the *labeled circles* (called “places”) represent *conditions* that can be true or false, and the *solid lines* (called “transitions”) represent decision making: if all the input conditions are true, the transition is “fired.” The result is that the conditions in the input places are put to false again, and those in the output places become true. The truth values of the “source” places (i.e., those without input transition) are *determined* by the flags in the *protocol* arrays. Similarly, the truth values of the “sink” places (i.e., without output transition) *determine* the value of the corresponding flags in the *protocol*.

The ideal lifetime of an event is “zero”: as soon as an event is *fired* by an activity, all the activities that need to react to the event (that is, “to handle” it) will *consume* the event during their reaction. The software architecture of such coordinated components must foresee the *communication* of events between the firing activity and each of the handling activities, which is (one of the reasons) why asynchronous data exchange is needed between these activities.

Figure 1 uses the simplest form of a protocol sequence, namely, an *array*; in general, protocols consist of compositions of more than one such array, representing different allowable “paths” in the coordination. Note the important difference between the very narrow and lean semantics of a “protocol” as needed in this paper and the much wider semantics of “protocol stacks” as used in inter-process communication (Delanote et al., 2008).

### 1.3 Archetypical use cases

The following example set of multi-robot applications, with multi-tasking functionalities for each robot, is representative of the scope of this paper’s coordination design contributions:

• Workspace sharing. This involves scenarios where multiple mobile robots (flying, wheeled, and legged) from possibly different vendors (and hence with independently developed software capabilities) need to share the same space in a warehouse or orchard. The same holds for multiple manipulator arms on conveyor belts or at assembly and fruit harvesting stations. In addition, both types of robotic components should also physically interact with each other, like an assembly robot arm that can take parts from a mobile robot that brings the parts from storage.

• Execution protocols. For example, robots must *register* with the “manager” of a shared resource (charging station, parking space, inland waterway lock, and gripper on a fruit harvesting robot) and then follow a *protocol* coordinated by that manager every time they want to use that resource. Being able to coordinate the execution of different robots in a predictable, agreed-upon way is another necessary (but not sufficient) condition for sharing *physical workspace.*

• Task sharing**.** A typical example is two mobile robots in a manufacturing cell that coordinate how to share the same *areas* during the execution of their tasks, such as driving the *routes* through the depicted stations. Other applications requiring robots to share task executions are include carrying or pushing a shared load, covering a whole agriculture field or a surveillance area, closing a control loop around other robots’ sensor capabilities, and platooning in traffic. Task sharing is *the* driving *end-user pull* behind having to spend design and implementation efforts on all the archetypical challenges mentioned above.

### 1.4 Scope

This paper focuses on the software design of the **coordination** of *runtime decision making*, including data structures, policies, decision-making functionalities, software patterns, and best practices. An implementation using Petri nets, with the purpose of being used within these coordination patterns, is explained and evaluated. As the final validation, the previous patterns are applied to two coordinated navigation cases.

Subjects outside the scope of this study are the *functional algorithms* that define the *behavior* inside activities, the creation of *maps* and *Petri nets*, the *policies* behind the reasons why the *application* takes these decisions, and the *communication functionalities* via which activities exchange the *data* they need from each other to realize their functional behavior.

### 1.5 Contributions

The *contributions* of the paper are

• The software mechanisms of coordination, which encompasses everything needed to fire and handle events that allow concurrent activities to coordinate their executions. In particular, this includes the complementary roles of *finite state machines* and *Petri nets* by introducing two non-traditional primitives (the *protocol array* and the *event circular buffer*) that help in the *separation of concerns* (Dijkstra, 1982) of the mentioned complementary roles within the presented software design.

• Explicit awareness of the implementation constraints, which are introduced by the distributed, multi-core computer infrastructure common in modern robotics applications. In particular, this includes ensuring event data consistency between concurrent activities via *circular buffers* and optimizing execution efficiency by exploiting *data locality* and *cache awareness.*

## 2 Related work

The coordination of components is only one of the necessary “concerns” that large-scale “cyber–physical” systems must deal with. It fits into the broader context of the “5Cs” approach of making systems-of-systems software architecture (Bruyninckx, 2023; Klotzbücher et al., 2012; Radestock and Eisenbach, 1996; Vanthienen et al., 2014). The five parts of the 5Cs meta model are

• Computation: the functional behavior inside each activity.

• Communication: the data exchange behavior between activities.

• Coordination: the decision making behavior in and between activities.

• Configuration: adapting each activity’s behavior to the actual context.

• Composition: the integration of the previous four parts at the “levels” of activity, component, system, and system-of-systems architecture.

Each of the first four “Cs” can, in itself, be a full or partial sub-system of the “5Cs”. A very established pattern within the coordination “C” is that of the life cycle state machine (LCSM), responsible for the “top-level” coordination *inside one single activity*: to create, to start up, to execute, to pause, to reconfigure, and to shut down activities (and the resources they manage) in predictable and composable ways. One single robot will have many activities (sensing, control, world modeling, task execution, etc.), each with its own LCSM, and the focus of this paper is to explain how to maintain the coordination between all these LCSMs, which is where the Petri nets come into play.

Petri nets have been widely used for *modeling concurrent activities/processes* (e.g., to analyze the concurrency behavior of several activities with respect to deadlock analysis or reachability analysis), and their *implementations* come in various forms depending on the use case context in which they are deployed. The implementation proposed by Davidrajuh (2010) has been widely used with MATLAB integration for Petri net modeling, simulation, and performance analysis. In the case of generalized stochastic Petri nets, the implementation proposed by Dingle et al. (2009) provides an open-source tool for design and analysis. The TINA toolbox (Berthomieu et al., 2004) offers a broad set of tools for the construction and analysis of Petri nets and timed Petri nets, which has been extensively used in academia. IOPT-Tools (Pereira et al., 2022; Gomes et al., 2010) provide a framework for the automatic generation of controller code from a modeled Petri net. Developments toward the implementation of Petri nets for microcontrollers have been researched by Kučera et al. (2020), providing a framework to model timed interpreted Petri nets to be used in Arduino devices.

While these implementations provide frameworks to work with Petri nets for different purposes, they are not focused on optimization for low-latency execution. This focus is a primary motivator for the research presented in this paper because modern robotic applications must coordinate several activities such as control, perception, world modeling, and task monitoring, many of which expect *real-time determinism* (Abdellatif et al., 2013). Piedrafita and Villarroel (2011) analyzed the execution dynamics of four different Petri net *software* implementation techniques, whose performance is evaluated with the same Petri net models as in this paper.

For robotics applications, Ziparo et al. (2011) used Petri nets as models for multi-body and multi-robot execution and planning. Their modeling within a multi-robot context is analyzed by Costelha and Lima (2007), investigating deadlocks and reachability. Figat et al. (2017) and Figat and Zieliński (2022) focused on, respectively, hierarchical finite state machines and Petri nets. Zhou et al. (2017) used a hierarchical FSM for the control of a navigation base with a manipulator, where one FSM is embedded into a higher FSM. Lacerda and Lima (2019) generated Petri nets for the coordination of a fleet of robots according to the time logic constraints of the coordinated execution.

## 3 Methodology

The focus of this paper is on three of the “5Cs” software concerns:

• *Coordination* : managing the interactions between a (possible large) set of concurrently executing *activities* using flags, events, finite state machines, and Petri nets as the sufficient mechanisms.

• *Configuration*: allowing application developers to steer the *execution efficiency* of their applications: 1) the *pre-processing* of data structures used by the coordination primitives at runtime and 2) the *event firing and handling* mechanisms that each *coordinated activity* needs to interact with the *coordinating activity.*

• *Communication*: facilitating the exchange of *events* between the finite state machines in the *coordinated activities* on the one hand and the *coordinating mediator’*s Petri net on the other hand.

In addition to the *separation of concerns* (Dijkstra, 1982) that already come with the “5Cs” approach, this paper adds other separations of concerns pertaining to the design of the *inside* of the relevant “5Cs” components. More concretely, the design of the *data structures* and *operators* needed to implement the envisaged coordination mechanisms.

### 3.1 Coordination mechanisms

The mechanisms needed for the coordination of activities are conceptually very simple: *flags*, *events*, *Petri nets*, and *finite state machines* (Section 1.2).

A *finite state machine* (Hrúz and Zhou, 2007; Mealy, 1955) models the *discrete behaviors* of one *single activity*. Its four data structures are the *sets* of 1) *states* that the activity can be in, 2) *transitions* that are allowed between states, 3) *events* that can trigger *transitions*, and 4) *flags* whose status is linked with (a subset of) the *events*. The latter is added to the *mathematical* representation of an FSM in Section 1.2 to allow the *interaction* between an FSM and a Petri net. Its functions are 1) to process the list of available *events*, 2) to compute which *transition* each of those events will trigger (when processed in order of arrival), and 3) to adapt the above-mentioned data structures accordingly.

**FIGURE 2:**
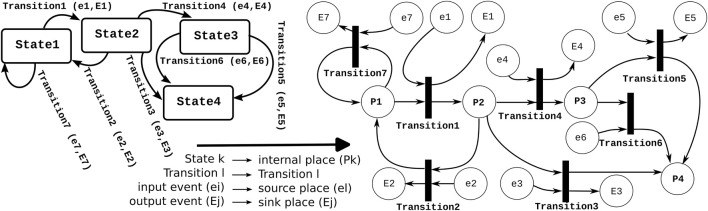
A *finite state machine* and the mapping to its equivalent *Petri net*. This mapping constrains the Petri net to have only one connector between any *internal place* and the transitions connected to that place. All other places map to “sink” or “source” events; the “source” places are denoted with small letters, and the “sink” places are denoted with capital letters. A similar typographical convention is used for input and output events in the finite state machine.

From a *software implementation* point of view (but *not* from a semantics point of view), finite state machines are just a boundary case of Petri nets: the former has a constraint on the number of “tokens,” namely, *exactly one* in the whole set of “states.” Figure 2 shows an example of the mapping of an FSM to an equivalent Petri net.

So, this paper focuses on the software design of Petri nets because that of finite state machines differs only in the *configuration* of the resulting library and the *naming* of the implementation primitives. A Petri net model shares the four above-mentioned building blocks with a finite state machine model, but it uses the following specific terminology: a *place* that can contain zero or one *token* as a *marking*, a *transition*, and a *directed arc* between them. The constraint on an arc is that its start and end must be either a place or a transition; in other words, places are only connected to transitions and vice versa. The constraint of a maximum of one token per place is what Murata (1989) referred to as “finite capacity nets of capacity one for all places”; other works of literature call it “safe Petri nets” (Barylska et al., 2017). A *transition* represents a coordination point in the Petri net: its *input places* represent the conditions to be fulfilled for that synchronization to take place; and its *output places* represent the status changes triggered by the coordination.

In addition to the above-described *data structures*, the Petri net mechanism also has some *operators* (“behavior”) on these data structures. If each input place of a particular transition has a token, that transition is *enabled*, and *firing* a transition implies that the tokens in its input places are *removed* and the tokens in its output places are *filled*. The token in the *source places* is to be filled by the processing of an *event* that comes from “somewhere.” Similarly, removing a token from a *sink place* gives rise to sending an event “somewhere.” The links with that “somewhere” are discussed in the following section on “communication.”

**FIGURE 3:**
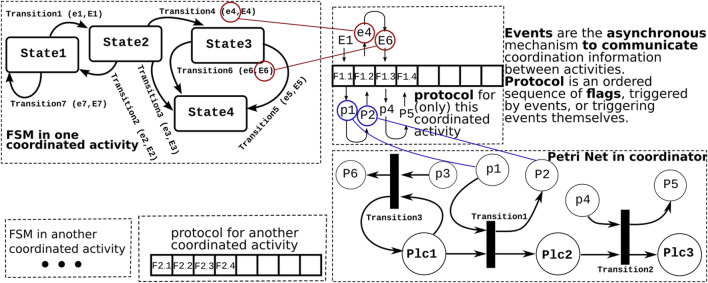
Examples of the three software mechanisms needed in *interactivity coordination*: A Petri net inside a *coordinating* activity, a finite state machine inside each of the *coordinated* activities, and (an array of) flags for the bookkeeping of which *coordination* “*events*” have been communicated between both. Capital letters are used for *output events* in the finite state machine and for *sink places* in the Petri net. The colored lines link events and places to locations in the protocol array. The “snake-like” trajectory through the array represents the temporal order in which the “communication” takes place between finite state machine events and the marking of places in the Petri net.

Notably, in Figure 2, the FSM and Petri net represent the same process; however, throughout the paper, this is not the case. FSMs are used for the discrete behaviors of single activities, while the Petri nets are used for the coordination across activities. This means there is a match among the FSM states of the coordinated activities and Petri net places of the coordinator; however, they do not present the same process. The latest is illustrated in Figure 3.

### 3.2 Communication mechanisms

The finite state machine in each of the *coordinated activities* exchanges *events* with the *coordinating mediator*’s Petri net (Figure 3). This is reflected in the structure of the Petri net as follows:

• Some input places of transitions do not have any transitions for which they are output places, e.g., p1, p3, and p4 in Figure 3; these are called *source places.* Source places are filled in by the arrival of events to the owner of the Petri net activity. In Figure 3, a token is added to source place p1 when external event 1 (E1) is processed.

• Similarly, *sink places* do not have any transitions for which they are the input places, e.g., P2, P5, and P6 in Figure 3. Sink places trigger the sending of events from the owner of the Petri net activity to other connected activities. In Figure 3, sink place P2 causes the triggering of the internally generated event 4 (e4).

Source and sink places are the locations where the Petri net is connected to *events* from and to the “outside world.” *Internal places* are all other places.

The contribution of this paper with respect to *communication* pertains to the introduction of the *protocol* data structure: it *decouples* the *internals* of the finite state machines and Petri nets from the *communication* of the information they need for their coordination.

The protocol contains information regarding which of the two activities involved in the coordination is expected to set the next *flag* in the protocol. This document uses *arrays* as protocol data structures since they are the simplest approach needed to realize the following goal:

• Only those events that a coordinated activity or the coordinating activity generates or reacts to “end up” in the protocol data structure. These are the events that need to be shared between them.

• The protocol introduces a *hard constraint* in the order in which these events are allowed/expected to be generated; Figure 3 represents this order by the “snake-like” trajectory through the protocol data structure. In order *to guarantee* the correct execution of the coordination, both coordinated parties must satisfy these hard constraints in the *sequence* in which the relevant events are generated or reacted to by the finite state machine and in which the sink and source places are marked in the Petri net.

A flag can be set directly by an activity, or it is the result of processing an event received from that activity. Because of the strict order brought by the protocol, there is no risk that this asynchronous access to the data introduces inconsistency.

### 3.3 Configuration mechanisms

This section introduces three software patterns that provide the mechanisms needed *to configure* the coordination between activities. The patterns themselves are not explained in detail because that part of the authors’ research is beyond the scope of this document. However, they are in use in the experimental demonstration in Section 5. Each of these patterns works at a different *time scale* in the coordination interaction:

• *Semantic registration* (“long term”): an activity that needs to be coordinated is registered (by itself or by a “third party”) for a particular coordination using a *semantic ID.* This ID is a *symbolic* unique identifier used in a *model* of the coordination and, hence, can be retrieved from *persistent storage* or *inter-process communication.*

• *Symbol table data structure* (“medium term”): it links the *semantic ID* symbol to a (possibly variable) number of “resources” or “components.” The table facilitates the discovery, communication, execution, and introspection of the “resource” at runtime, which can also be done by activities that have been developed independently.

• *Acquire–release* (“short term”): this pattern structures access to a shared resource by expecting the resource-using activities *to acquire* access from the resource-owning activity and *to release* their granted access explicitly.

The *registration* puts the semantic ID into a table (or a “map”) with (at least) the following columns:

• The *semantic ID.*

• The *name* of the coordinated activity, as used in the *source code* of the implementation.

• The *binary pointer(s)* to the memory where the coordination data structure(s) are stored.

Table 1 shows an example of such a symbol table. The semantic ID itself has two fields, datatype and model. There can be multiple semantic IDs with the same model label, but the tuple (datatype, model) must be unique. Multiple activities can access the same variables, and coordination is done via mutexes.

**TABLE 1:** Example of a table for *registering* the access of activities to shared resources. This particular example uses a *mutex* to coordinate the access to data structures encoder_t and motor_t, shared by three activities in a robot, control, proprioception, and drive.

Semantic ID				
Datatype	Model	Pointer	Mutex	Activity
encoder_t	Left	0x0a00	0x0a08	Drive and proprioception
encoder_t	Right	0x0a10	0x0a18	Drive and proprioception
motor_t	Left	0x0a20	0x0a28	Drive and control
motor_t	Right	0x0a30	0x0a38	Drive and control

The above-mentioned mechanisms are needed for the following reasons:

• *Unambiguous ownership*: registration *implies* that there is a “shared object” to register to and that the system developers should make one, and only one, *activity* the responsible “owner” of that object. (The “owning” object can be a fully passive library and need not be an activity in itself.)

• *Runtime reconfiguration*: because registrations are objects with a lifetime, they can have a life cycle state machine on their own. This is important to coordinate the reconfiguration of the “object” at runtime and between a changing number of registered activities.

This paper focuses on this short-term time scale, hence, on a low-latency implementation of the *acquire–release* protocol. The “objects” in this paper are coordination objects, specifically Petri nets, and the scope of the presented research calls for Petri nets to be created at runtime. For example, in manufacturing or logistics cases, dozens of shared resources occur, to which, at *any time*, two, three, or more robots want access, and those robots can be different ones every time.

## 4 Implementation

The focus of the paper is on the software mechanisms that are used to realize coordination between a (possibly large) set of concurrently executing activities. The Petri net model plays a central role within the coordination mechanisms presented in the last section. Therefore, an implementation with the purpose of multi activity coordination is presented.

This paper’s *design drivers* of the *implementation* of the *design* discussed in Section 3 are typical for *embedded* systems: low-latency and asynchronicity within a shared memory deployment. The presented design is not claimed to be efficient for other use cases, such as the *offline analysis* of Petri nets in search of deadlocks, livelocks, starvation, etc.

One implementation decision is easy to make: while *finite state machines* and *Petri nets* are two complementary coordination mechanisms at the *conceptual* level, their *implementations* are extremely similar; both need “states” and “transitions,” with incoming “events” as triggers of the evaluation of the mechanism, as well as the evaluation’s possible outcomes. Figure 2 explains the direct mapping of a finite state machine into the equivalent Petri net, so this section restricts itself to the implementation of Petri nets only.

This summary from previous sections is behind the other implementation decisions:

• Petri net models are expected to be *generated at runtime* from symbolic *models.* This allows the use of data structures that can exploit the knowledge of the *number* of *places*, *transitions*, and *events.*

• Petri nets are expected to be *executed in an event loop* of *real-time* activities (Samek and Ward, 2006). This allows a “5Cs” design that *pre-empts* the execution when a *maximum number* of transitions, places, and/or events have been processed, with a known impact on the latency this introduces.

• The *coordinated activities* typically run *asynchronously* with the *coordinating activity* (that is, the one that executes the Petri net). Hence, measures have to be taken to guarantee *data consistency.* This implementation provides two of these measures: *memory barriers* with acquire and release semantics (Standardization committee C and C++, 2017) and *circular buffers* for *wait-free* exchange of events (Desnoyers and Dagenais, 2012; Varghese and Lauck, 1987).

• The target applications are expected to be *always on*, so all of the above-mentioned features must be *(re)configurable.*

### 4.1 Data structures

**FIGURE 4:**
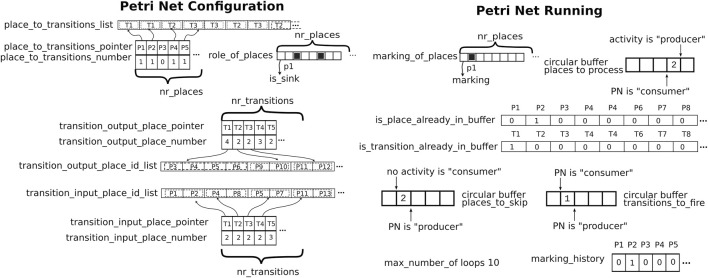
Overview of the data structures used in this paper’s implementation of Petri nets. Left: *to represent* a Petri net. Right: *to execute* a Petri net. Both sets can be *(re)configured* at compilation time or runtime.

Figure 4 shows the data structures to represent and execute Petri nets. The data structures above will always be accessed *synchronously* within only the *Petri net executor* activity. The efficiency is designed for the following execution use case:

• Computation of the status changes: the Petri net’s status is updated as soon as the activity reacts to incoming events. The events are received asynchronously by the Petri net executor activity (in the *communication* part of the activity’s event loop, Supplementary Appendix SA), and our design uses *circular buffers* for this purpose. Circular buffers are also used inside the synchronous part to encode the “to-do lists” of places and transitions that need processing based on the incoming events. The buffers make use of *memory barriers* (of the *acquire-release* type, as provided by the *concurrency support* part of the C/C++ standard libraries) in the trade-off between efficiency of execution and the consistency of data. The latter is a concern to be dealt with by the *application* developers and is introduced by *out-of-order execution* optimizations in modern compilers and CPUs.

• *Data locality*: the data structures needed in nearby moments in the computations are stored in nearby bytes in physical memory. So, *cache coherence* is optimized in two complementary ways:

• Minimally sized data structures to keep their status. For example, when there are N places, one needs only M 8-bit bytes, where 8×M is the smallest number larger than N . For example, when there are less than 255 places in a Petri net, one char is enough. Such low numbers are not exceptional in the use cases of this paper because access coordination is almost always very local and between a low number of coordinated activities.

• All data structures are *arrays* of the same type. This reduces the need for *padding* between non-homogeneous parts in the data structures and, hence, indirectly their size as well.

• The individual data structures are all *cache line aligned* to avoid *cache trashing.*

• *Arrays instead of linked lists*: the *semantic IDs* of the representation of places, transitions, and events are mapped to unsigned integers ranging from 0 to an *a priori* known integer value N . These integers can then also serve as *indices in arrays* so that the inefficient search through lists is replaced by efficient direct access into the arrays.

This section uses teletype font, like this, to represent data structures and operations that are used in the software implementation of this paper’s concepts. The following data structures represent the *structure* of a Petri net (Figure 4, left):

• place_to_transitions: this is a *map* (or *symbol table* or *associative array*, Section 3.3) to quickly find the *output transitions* of a place with a given ID. It contains i) pointers bi in an array place_to_transitions_pointer to the binary representation of the transition with a given ID and ii) an array place_to_transitions_number containing the number of transitions for the referenced place.

• transition_input_place and transition_output_place: similar to place_to_transition, these maps allow quick access to the output and input places of a given transition.

• sink_places: an array of bits in which each bit represents whether the place is a sink. There is no need to encode whether a place is a *source* or an *internal* place as their behavior does not impact the synchronous execution of the Petri net.

The following data structures represent the *synchronous execution status* of a Petri net (Figure 4, right):

• marking: similar to sink_places, this bit array encodes which places are marked and are, hence, candidates to be processed next.

• places_to_process: this circular buffer (fully inside the *synchronous* context of the coordinating activity) represents the *to-do* list of the IDs of places that must still be inspected to detect *enabled* transitions. In addition, the size of this array can be kept minimal, given the knowledge of the number of places. It also does not make sense to put one particular place more than once on this *to-do* list.

• places_to_skip: this circular buffer represents the list of the IDs of places that still have to be processed, but whose processing has been postponed until the next run of the event loop. Because of the *event loop* context and the *deterministic low-latency* driver, the system developers can decide to limit the number of places on the to-do list that will be processed in each run of the event loop and the number of times such processing is done. This approach provides a *configurable trade-off* between reactivity and deterministic execution time via the configuration variables below.

• is_place_already_in_buffer: these bit arrays remember whether a place is already being checked to avoid the repetition of processing within the same execution loop.

• marking_history: this array of L unsigned integers contains counters indicating how many times each place in the Petri net *has been processed* during this event loop execution.

• max_number_of_loops: this defines the maximum number of times a place *can be processed* per event loop execution before loop’s execution is preempted.

• transitions_to_fire and is_transition_already_in_buffer: these serve similar functions to places_to_process and is_place_already_in_buffer but are used for processing of transitions instead of places.

In order to reduce the *cache missing* latency when accessing all these data structures, they should be aligned on cache lines, including padding the last needed cache line with empty bytes.

### 4.2 Discussion

The presented design aims to improve execution latency at the cost of some extra memory in the data structures:

• The data structures place_to_transition and transition_input_place *both* encode the connection of outgoing arcs from places to transitions of the Petri net. This redundancy in memory allows faster lookups in the Petri net execution loop.

• The IDs to process in the circular buffers transitions_to_fire and places_to_fire correspond one-on-one to the flags marked in the status buffers is_place_already_in_buffer and is_transition_already_in_buffer. Every time a new entry is added to the circular buffers, it is also added to the status buffers. This is, strictly speaking, redundant information, but this redundancy yields fast verification of what is already in the *to-do lists*, hence avoiding repeated processing of the same data.

• A similar motivation is behind the design of the data structures sink_to_events and sink_index, which also contain redundant information about the mapping from place ID to sink ID.

Installation instructions, examples, and the code for the implementation of Petri nets explained in this paper are available in^1^.

### 4.3 Results for generation and execution performance

To evaluate the execution time of the previous Petri net implementation, five Petri net models presented by Piedrafita and Villarroel (2011) have been built in the library. The range for scaling the size of the Petri nets is taken from the same reference. The Petri net models built were as follows:

• SEQ: Petri nets of *p* sequential processes.

• PR1: Petri nets of *p* sequential processes with two states and one shared resource.

• P1R: Petri nets of one sequential process with *p* resources.

• PH: Petri net of the philosophers’ problem with *p* philosophers.

• SQUARE: Petri nets of *p* sequential processes with *p-1* resources.

Within this context, two tests were performed: 1) performance test with immediate firing of one transition; in this case, the execution time of 2,000 triggered transitions is measured. 2) Test with immediate firing of all transitions in the net; this type of test is expected as it marks the maximum reaction time for the complete evaluation of the Petri net. In the latest test, all the transitions of the Petri net will be enabled and triggered in each loop as the Petri net is saturated. The execution time is measured for 2,000 loops for each Petri net. The tests have been run on an HP ZBook Firefly 14 G7 Mobile Workstation.

**FIGURE 5:**
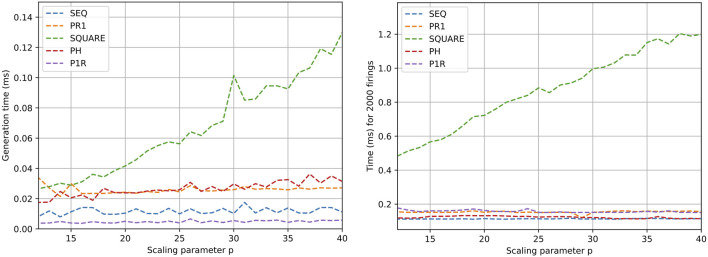
Generation (left) and execution (right) time results for different Petri net models. Execution time refers to transition triggering in the net 2,000 times.

**FIGURE 6:**
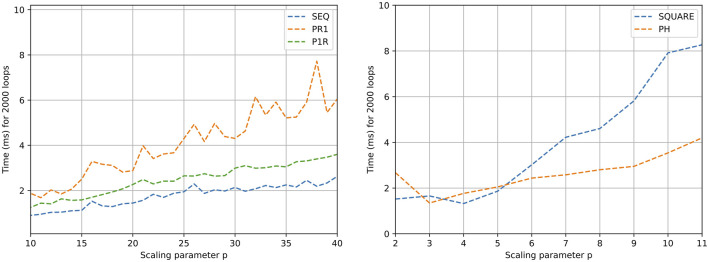
Time results for different Petri net design executions. Execution of all transitions enabled in the net 2,000 times.

The X-axis in Figures 5, 6 marks the computation time, while the Y-axis is the scaling parameter, which denotes the number of sub nets in the Petri net [as described by Piedrafita and Villarroel (2011)]. Figure 5 (left) presents the generation time for the Petri nets. The generation time comprises both memory allocation for the data structures in Figure 4 and its initialization. For the Petri net models SEQ, PR1, PH, and P1R, the allocation time is dominant over the initialization time, making the generation time stable within the order of nets tested. In the case of SQUARE, as the size of the net scales quadratically, the initialization time dominates.

Figure 5 (right) shows the performance of the execution of firing one transition per Petri net evaluation. In the case of SEQ, PR1, PH, and P1R, the execution time does not escalate with size, as the number of filled outgoing places from transitions is constant. In the case of SQUARE Petri nets, as the scale factor increases, the number of places to be filled after triggering a transition increases proportionally. Figure 6 shows the execution of saturated Petri nets. The execution time grows linearly for the Petri net models SEQ, PR1, PH, and P1R. This is expected because the number of evaluations is proportional to the number of places in the net. With the same logic, the time for the SQUARE Petri nets grows quadratically with respect to the scale parameter.

As the Petri nets are saturated in the second set of tests (Figure 6), the time in the graphs is taken as an upper bound for the processing time of the Petri net. For instance, a sequential Petri net with 20 processes can take up to 722 ns (1.44 ms/2,000) in the case of all processes coordinated from a mediator.

## 5 Experimental validation

The design and best practices proposed in this article were applied in an experimental setup with two autonomous mobile robots (AMRs) operating in an area with a pre-defined traffic layout. The demonstration case is an artificial scenario of an emergency AMR entering an area with an AMR operating at a lower speed. According to the situation, the slower robot has to reconfigure its execution at discrete and continuous levels in order to let the emergency AMR overtake. Moreover, for the coordination in the shared area, a mediator is introduced to ensure the execution of the synchronization of the AMRs.

### 5.1 Robot setup

**FIGURE 7:**
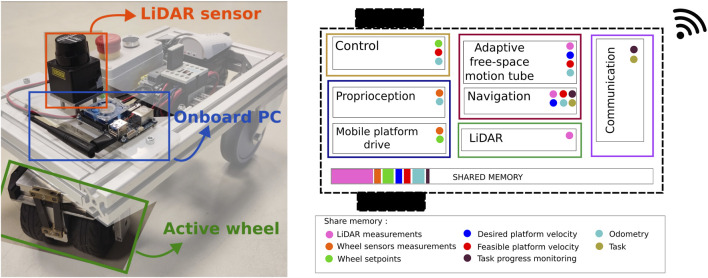
Mobile platform, hardware view (left), and thread and activities running on the onboard computer (right). Activities running in the same process exchange data via shared memory. Some of the data chunks accessed via shared memory are illustrated in small colored circles.

Figure 7 shows one of the identical mobile platforms and the 5C activity components running on the onboard computer. Each platform is equipped with an active KELO drive 100, a Hokuyo URG-04LX LiDAR Sensor, and an ODROID XU4 Embedded Computer. In each robot, the following activities are running:

• *Mobile platform drive*: this receives sensor data and transmits wheel setpoints via EtherCAT to the KELO wheel drive.

• *Proprioception*: this estimates the relative motion of the vehicle using wheel encoders (dead-reckoning).

• *LiDAR*: this captures range data via a serial interface from the Hokuyo URG-04LX.

• *Navigation*: this detects and tracks features in the environment (perception) and computes the steering and forward speed commands to perform a desired maneuver (control).

• *Adaptive free-space motion tube*: this evaluates and adapts the control commands provided by the navigation activity to ensure that the vehicle moves within the free-space.

• *Control*: this transform control commands (steering and speed) to KELO wheel setpoints.

• *Communication*: exchanges data with other processes.

These seven activities are registered in five threads (represented in different colors in Figure 7) running at different frequencies. The five threads run in a single multi-threaded process, which allows for efficient in-memory data exchange among the different components. Figure 7 shows some examples of data shared between the activities in colored circles. For that, the variables (objects) need to be first registered in the symbol table with a semantic ID (name and datatype) by the activity owning the resource, e.g., “LiDAR measurements,” range_scan_t is registered by the LiDAR activity. For access to a shared variable, first, an activity requests the data pointers corresponding to a particular semantic ID (configuration) from the symbol table. After that, it can read the values (using acquire/release) directly from the memory without going through the symbol table (communication).

**FIGURE 8:**
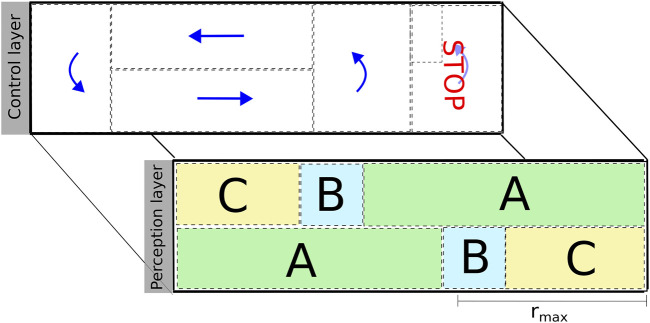
Illustration of the control and perception layers of the semantic map designed for the experimental validation. These layers encode the expected control and perception behaviors of the robot within a particular area. Both layers have several monitors associated with them for triggering the coordination mechanism and reconfiguring the schedule of the navigation activity of the platforms.

The traffic layout consists of semantic areas that are anchored in environmental features perceived by the robot (corridor and dead-ends). Figure 8 shows the control and perception layers of the semantic map. A solid black box around the map indicates solid walls detected by the LiDAR, while dashed black lines limit semantic areas in each of the layers. The control layer indicates the maneuver that a robot is expected to perform: move forward, make a U-turn, or stop. It also encodes constraints such as limits for driving velocity and deviation from the lane. The perception layer shows the feature that the robot has to track in different colored rectangles labeled as “A,” “B,” and “C.” For example, in area “A” (green), the robot resorts to a corridor detection and tracking algorithm for estimating its relative orientation and lateral position with respect to the corridor. In area “C” (yellow), the robot also tracks its relative longitudinal position with respect to the end of the solid wall at the end of the lane. The reason for the different perception behaviors is due to the finite range of the sensor, which is limited by $r_{\max}$ . The robot does not continuously search for the solid wall at the end of the lane but only when it reaches area “B” (blue).

The schedule of the navigation activity links together perception, control, and monitoring algorithms in the form of a skill. The schedule of the activity changes at runtime according to the situation due to coordination and (re)configuration. For example, the robot starts in a known location of area “A” and moves around the circuit. A monitor that uses the information provided by dead-reckoning detects that the robot has reached area “B.” The schedule of the navigation activity changes: the algorithm for detecting the end of the lane is added to the schedule, along with a monitor that checks whether the quality of the estimation is stable. When the estimation is stable, the schedule of the navigation activity changes once again by adding (end-of-lane controller) and removing (corridor controller) algorithms accordingly.

### 5.2 Coordinator setup

For coordination purposes in the semantic area, an area manager is introduced for registering robots in an area and sending events to the robots when necessary. These events will trigger the (re)configuration of the schedule of the robots. The area manager is a multi-threaded process running on a different computer. It has two activities composed according to the 5C paradigm:

• *Area management*: this keeps track of the coordination state of the area and coordinates the robots if required.

• *Communication*: this binds and starts the communication with the robots. It is connected through shared memory to the area management activity. It shares a queue with the updates (task progress monitoring) from the robot and sends commands (tasks) from the area management activity in its event loop.

In this experiment, the execution of commands from both robots is not coupled, meaning that the autonomous execution of each robot does not implicitly change according to other robots in the area. Instead, the area manager works as a mediator between the two robots, coordinating them.

• The area manager makes the decisions on the interaction behavior: the interaction of the bases with their shared resource (space) is set by the mediator, giving access to the areas it manages through events.

• The area manager decouples execution: the area manager is the only “agent” aware of the complete state of the coordinated execution at the discrete level by keeping the execution state in a Petri net.

• The area manager allows execution when the robots have incomplete information of the environment: the robots do not detect each other in the experimental setup proposed, which means that the mediator is required to allow the execution in shared spaces without disruption.

There are two acquire–release protocols between the area manager and AMRs. One of which is from the robot to the area manager to access the area. When the robot is navigating toward a local area, it has to request access to the area manager. When access is granted, the semantic ID of the robot and its role (normal or emergency robot) are registered in the list of robots coordinated by the area manager. This list contains the robots that “own” the area (as a passive resource) at a given time.

The area management activity coordinates the interaction of the robots in the area via the following components:

• Area state: the record of the robots that have requested entering an area and releasing an area. In case two robots declare they need to enter the same area, the area management activity sends control requests to the robots.

• Petri net state: when coordination among the robots in the area is required, a Petri net model with the coordination in the area gets initialized. On top of the coordinated states in the Petri net, configuration parameters can be added.

**FIGURE 9:**
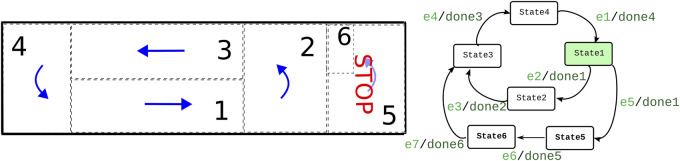
Finite state machine of AMRs and navigation map. The states in the finite state machine denote the traversal of the numbered areas according to directional arrows in the map. For example, state 1 would be the navigation in area 1. The input events (light green, at the left-hand side of the slash) among states come from either the navigation or communication activity (from the area manager). The output events (dark green, at the right-hand side of the slash) are triggered by the navigation activity.

**FIGURE 10:**
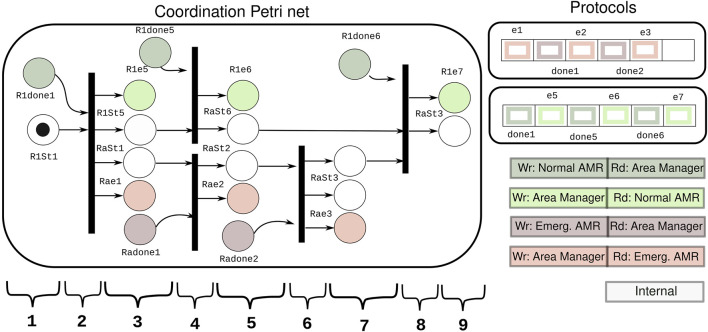
Petri net and protocols used for coordinating the normal AMR and the emergency AMR in the demonstration. The color legend shows the reading and writing “rights” for the flags in the protocols. The execution of the coordination at the discrete level according to the coordination in the Petri net: 1) normal AMR crosses area 1. 2) Wait until normal AMR crosses. 3) Emergency AMR is allowed to start its execution in area 1, while normal AMR gets the signal to go to the padding area with higher speed. 4) Wait for normal AMR to be out in area 5 and emergency AMR finishes in area 1. 5) Normal AMR is in padding area while emergency AMR crosses area 2. 6) Wait for emergency to be done in area 2. 7) Emergency AMR can start crossing area 3, and it is released from the coordination. 8) Wait for the normal AMR to go out of the padding area. 9) Normal AMR can start crossing area 3, and it is released from the coordination.

When coordination is needed among the robots in a given area, a second acquire–release interaction is established. The area management activity “acquires” the discrete control of the AMRs and releases it when the coordination is over. This means that, while the robot normally coordinates itself by executing the skills in its FSM depicted in Figure 9, when coordination happens, this is not the case anymore. When coordinated, the management activity takes control of the AMR at the discrete level via the coordination Petri net (Figure 10), with its connected protocols that trigger the sending of events to the AMRs. While the FSM in the robot is still tracking the execution of the robot, it does not trigger the maneuvers. When the coordinated execution is finished, the area management activity releases the AMR execution with a last shared event and deletes the coordination.

The Petri net used in the demonstration is depicted in Figure 10. The color legend of the image explains the ownership of the places, meaning which activity has control over the events to set the marking in the place. The white places are internal places of the Petri net, which denote the state of the AMRs in the execution. The dark places are source places, which are filled in by the coordinating activity once the corresponding event arrives from the AMRs. The light places are sink places to be filled in by the Petri net execution, triggering the sending of events to the AMRs.

For example, Figure 10 shows a case of the sink place “Rae1,” to which only the area manager has writing access. Its marking is filled in by the triggering of the Petri net. When the place “Rae1” gets a token, the connected flag “e1” in the protocol with the communication activity is raised. When the flag “e1” is raised, an event is sent to the emergency AMR, which indicates it may enter “Area 1.”

A case of the source place is “Radone1.” The communication with the emergency AMR has writing access right to the flag “done1” in the protocol, and the area manager activity reads this flag. When the event arrives from the communication activity connected to the emergency AMR, the flag “done1” is raised. Once the flag “done1” is read by the area manager activity, the place “Radone1” gets a token, and the outgoing transitions can be evaluated to continue with the coordinated execution.

The processing of incoming and outgoing events according to the sources and sinks in the coordination structure of the Petri net in Figure 10 allows the execution of the coordinated motions of the robots without disruption. Moreover, apart from the sink and source places, the internal places are added to denote the concurrent state of different activities in the coordination.

### 5.3 Execution of experimental demonstration

The management activity remains idle after initialization until two robots are passed to it (along with a communication channel to them). The robots are passed according to their roles in the coordination: normal robot or emergency robot. The emergency robot has priority over the normal robot. The initial state of each robot is informed to the Petri net, which translates to the initial marking.

Once the coordination is properly configured, the event loop of the management activity starts. In the event loop, the management activity processes the messages coming from the AMRs, updating them on the execution progress with respect to the skill they are performing. The activity updates the marking of the coordination Petri net when the events of finished skills are received. After the marking of the Petri net is updated from all robots, the Petri net is triggered. In the case that all the places of a transition have a token, the marking of the Petri net is updated. The updated marking is then passed to the communication modules to send the events coming from the Petri net to the robots.

The area manager is the only activity aware of the coordinated execution but is not responsible for configuring the schedule being executed in the coordinated robots. The change in configuration (e.g., of the normal robot when the emergency AMR is behind) is achieved via events that are sent from the area manager to the robots via the communication channel. These events lead to a change in the configuration of the robots, e.g., emergency AMR needs to slow down because the normal robot is still ahead or the normal robot has to drive to area 5 and wait there.

Once the coordination has finished (the emergency has overtaken the normal robot), the area manager gives back control to the AMRs because there is no need for mediation. Both robots continue their autonomous execution with their initial configuration.

### 5.4 Secondary demonstration: area manager for heterogeneous AMRs

The same area manager as in the previous experiment was deployed in a setup with three heterogeneous AMRs. The demonstration case is the access area to docking stations in a warehouse. In this application, coordination is needed to mediate the access to an area that can be used as two lanes by two small AMRs or one lane for a big AMR. The execution of the coordinated robots and the Petri net used can be seen in one of the videos in the multimedia part of this paper.

## 6 Discussion

The paper’s focus is on the *efficient implementation* of runtime coordination needs in multi-robot applications. Most of the efficiency comes from *knowing in advance* (the sizes and types) of all data structures because that knowledge allows making the most cache-efficient and data locality-driven implementations: (almost) linear-time indexing of data pointers, optimal cache alignment, known maximum usage in both time and space, etc. These efficiencies are typically only possible and useful in *embedded* and/or *real-time* software systems. *Single producer*, *single consumer* event queues, and to-do lists are common practices in this context because it is normal “to know everything” about such systems.

This section discusses implementation decisions that system developers have to be aware of to make the best use of the presented design:

• *Semantic registration* of all primitives involved (activities, Petri nets, etc.): while all data structures and operators presented in this paper can be implemented manually from scratch, they are also designed to allow an even partial and gradual development path toward more *code generation*, from models of the coordination mechanisms to executable code. It is important to consider the most advanced version in the applications, i.e., the version in which these models are “downloaded” or “adapted” at runtime and updated code is generated by a running system itself.

• *Symbol tables* for data sharing: they facilitate data sharing among activities running concurrently. This not only helps the above-mentioned code generation but is also useful in allowing “browsing” or “introspection” of a running application: the symbolic names can then be used to navigate from “component” to “component” and inspect and/or adapt the local values in these components.

• *Acquire–release* pattern: this pattern is used for acquiring access to write and read the variables kept in the symbol table. At the deepest level of detail, the presented implementation already uses this pattern via the *acquire* and *release* semantics of memory barriers. However, a similar approach can be used at all higher levels of detail, such as to connect two robots to a third one at runtime, where the latter is responsible for the coordination of the unique access to one of its resources by the former two robots. For example, the third robot could let the first robot use its pan-tilt camera.

• The Petri net and finite state machine data structures need only to be known at the time of *runtime software configuration* (that is, not necessarily at *compile time* or even *deployment time*) because memory allocation can be postponed until just before the data structures are used. A *memory pool* approach is also a good fit.

• “*Best” design of the monitors.* Coordination is, by nature, a *reactive* behavior triggered by *events* that represent that “something has happened.” Hence, the efficiency and correctness of the coordination tasks in an application are not only realized by the efficiency and correctness of the coordination mechanisms presented in this paper but also by the “appropriate” design of the *monitors* that are needed to generate the events by monitoring Boolean combinations of status variables that can be spread over several components of the application. Similarly, the events generated by the coordination mechanisms must still be reacted to in an “appropriate” way via decision-making functions in the relevant system components.

• *Simultaneous events.* The application context of this paper is that of *concurrent activities*, each of which can generate multiple events and is expected to react to multiple events. No software design is known *to guarantee* that the *order* in which events end up in each activity’s event queue is the same temporal order in which these events were generated. Hence, the *system architects* have the responsibility to introduce coordination logic (in FSMs, Petri nets, and protocols) that is “*appropriately” robust* against such order “inversions.” To the best of the authors’ knowledge, the scientific foundations to generate such robust coordination logic are still to be discovered.

• *Errors in coordination logic.* Even a perfect software implementation of the mechanisms in this paper cannot guarantee that there are no errors in the coordination logic of the application, possibly leading to deadlocks or livelocks in the overall system.

For example, a robot might attempt to enter an area to which it has not yet been granted access to, or it might try to enter another area. System designs can be made more robust by introducing extra monitor activities to detect deadlocks or livelocks when the coordinated activities have not foreseen this monitoring themselves.

• *Hierarchy in coordination.* The coordination examples presented in this paper are “*flat*”: there is one Petri net layer added to the robots’ individual task controllers. This hides the implicit assumption that the coordinated robots collaborate *only* with the coordinating activity and that the actions that Petri net decides about have no “competition” of decisions made elsewhere. The authors believe that solutions to these problems are *not* to be found in extra software primitives but in using the presented ones in non-flat, application-specific “coordination hierarchies.”

One reason for introducing such “non-flat” coordination is to address the *coordination logic errors* of the previous item. In addition to the deadlock/livelock monitors mentioned above, system designs can be made more robust by introducing *pre-emption*: the Petri net and/or finite state machines are extended with places/states that represent a phase in the coordination where that coordination can be pre-empted. In any case, such pre-emption needs coordination itself because all coordinated activities must somehow be brought back into a known and consistent interaction state.

This “hierarchical coordination” topic is beyond the scope of this paper.

## Data Availability

The original contributions presented in the study are included in the article/Supplementary Material; further inquiries can be directed to the corresponding author.

## Footnotes

1
https://gitlab.kuleuven.be/u0141779/coordination_library.git
