# Supplementary material for: Software patterns and data structures for the runtime coordination of robots, with a focus on real-time execution performance
Source: Front Robot AI. 2024 Sep 4;11:1363041. doi: 10.3389/frobt.2024.1363041 (PMC11408128; doi:10.3389/frobt.2024.1363041)
Supplement: Supplementary file 1 [file Supplementaryfile1.pdf]

A APPENDIX: "5CS" COMPUTATIONS

This Section explains how coordinated and coordinating activities can be put into a multi-processing architecture, following the *5Cs meta model*, Bruyninckx (2023); Klotzbücher et al. (2012); Radestock and Eisenbach (1996); Vanthienen et al. (2014).

A Petri net is executed inside a *coordinating activity*, and as any other *resource* in the application, it has a *Life Cycle State Machine*, with the following states:

- `Create`: allocation of memory for the Petri net;
- `Configure resources`: initialization of Petri net and protocol arrays; registration of coordinated activities;
- `Configure capabilities`: switch `places_to_skip` and `places_to_process`;
- `Running`: consisting on executing `communication` and `computation`;
- `Delete`: de-registration of coordinated activities; deletion of Petri net and de-allocation of memory;

Moreover, the following *design invariants* are taken into account for the Petri net execution :

- there are no enabled transitions when the computations are started, and when they end.
- the order in which places and transitions are processed does not matter.
- the data structures are big enough to store the maximum number of entries that can occur.

For each protocol event that comes in, the *event loop* in the `Running` state of the coordinating activity executes the operations described in Algorithms 1, 2, and 3, in that order: 1) first a *communicate()* step where the incoming events are mapped to places, 2) the *computation()* as execution of the Petri net and 3) *communicate()* the events triggered by the newly processed sink places. The coordinating activity executes these operations in a synchronous manner.

The communication of events with external processes has to be conveyed by other activities within the same process. Therefore, the communication with external processes is handled asynchronously.

Event connection to places in the Petri net

Figure 1 shows the data structures for the `communication` in the *configuration* and *running* states of the Life Cycle State Machine of the Petri net coordination. That is, the basis for the handling of the events involved in the coordination. The data structures relevant for the *configuration* state of the coordination are:

- `event_to_source_pointer`, `event_to_source_number`: these are part of a map `event_to_source`, similar to `place_to_transitions`. to find the status data structure of a `source place` with a given `ID`, when an event with a given `ID` arrives through shared memory. This is needed in the protocol to convert external `events` to a source `place` with a given ID.
- `sink_to_events`: similar to the map `event_to_source`, to find the event(s) to fire when a *sink place* with a given `ID` is filled by the Petri net.
- `sink_index`: mapping from the *place* `ID` in the `place_transition` map to the corresponding `ID` in the `sink_to_events` data structure. If the place is not a sink, the respective `ID` is `NULL`.

The data structures relevant for the `running` state are:

- `sinks_to_events_counter`: similar to `marking_history`. Contains a counter on the number of times a *sink place* has been processed per execution of the Petri net.
- `sink_todo_list`: circular buffer with the *sink places* whose markings have been updated. The `ID`s of the `sink places` refer to the `sinks_to_events` map. From this information, the triggering of connected outgoing `events` starts.
- `events_to_process, events_to_communicate`: circular buffer with the incoming and outgoing `events` from and to other activities.

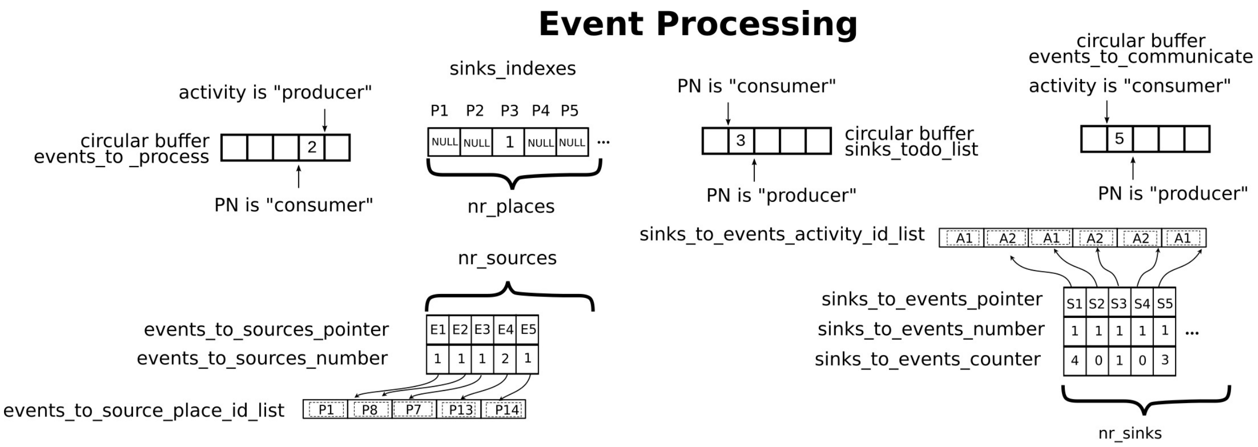

Figure 1. Data structures for the processing of events between asynchronously running coordinated activities, and the synchronous context of processing of events, places and transitions inside the Petri net's coordinating activity.

Algorithm 1 First *Communicate()*: get events to process from other activities

while $ID_{event} \leftarrow read\_next(events\_to\_process) \neq NULL$ **do**
  $places \leftarrow get\_event\_places(ID_{event}, event\_to\_sources\_map)$
  for ID_{place} in $places$ **do**
    $set\_marking(ID_{place}, marking\_of\_places)$
    $add\_new(ID_{place}, places\_to\_process, place\_buffer\_states)$
  end for
end while

REFERENCES

Bruyninckx, H. (2023). *Building blocks for complicated and situational aware robotic and cyber-physical systems*. Tech. rep., KU Leuven, Department of Mechanical Engineering

Klotzbücher, M., Biggs, G., and Bruyninckx, H. (2012). Pure coordination using the Coordinator–Configurator pattern. In *Proceedings of the 3rd International Workshop on Domain-Specific Languages and models for Robotic systems*. 1–4

Radestock, M. and Eisenbach, S. (1996). Coordination in evolving systems. In *Trends in Distributed Systems. CORBA and Beyond* (Springer-Verlag). 162–176

Vanthienen, D., Klotzbücher, M., and Bruyninckx, H. (2014). The 5C-based architectural Composition Pattern: lessons learned from re-developing the iTaSC framework for constraint-based robot programming. *Journal of Software Engineering in Robotics* 5, 17–35

Algorithm 2 *Compute()*: synchronous computation of Petri net

while $ID_{place} \leftarrow read\_next(places\_to\_process) \neq NULL$ **do**
  $remove(ID_{place}, place\_buffer\_states)$
  $increase(ID_{place}, marking\_history)$
  $transitions \leftarrow get\_transitions(ID_{place}, place\_to\_transitions\_map)$
  for $ID_{transition}$ in $transitions$ **do**
    $input\_places \leftarrow get\_places(ID_{transition}, transition\_input\_place\_map)$
    $enabled \leftarrow true$
    for ID_{input_place} in $input\_places$ **do**
      if $check\_value(ID_{input\_place}, marking\_of\_places) \neq 1$ **then**
        $enabled \leftarrow false$
      end if
    end for
    if enabled **then**
      $add\_new(ID_{transition}, transitions\_to\_fire, is\_transition\_already\_in\_buffer)$
    end if
  end for
  while $ID_{firing} \leftarrow read\_next(transitions\_to\_fire) \neq NULL$ **do**
    for ID_{input_place} in $input\_places$ **do**
      $remove\_marking(ID_{input\_place}, marking\_of\_places)$
    end for
    for ID_{output_place} in $output\_places$ **do**
      if $counter(ID_{output\_place}, marking\_history) \leq max\_number\_loops$ **then**
        $add\_new(ID_{output\_place}, places\_to\_process, is\_place\_already\_in\_buffer)$
        $set\_marking(ID_{output\_place}, marking\_of\_places)$
        if $check\_value(ID_{output\_place}, role\_of\_places) = 1$ **then**
          $add\_new(ID_{output\_place}, places\_to\_communicate)$
        end if
      else
        $add\_new(ID_{output\_place}, places\_to\_skip)$
      end if
    end for
  end while
end while

Algorithm 3 Second *Communicate()*: communicate events to other activities

while $ID_{place} \leftarrow read\_next(places\_to\_communicate) \neq NULL$ **do**
  $ID_{sink} \leftarrow get\_sink\_ID(ID_{place}, sink\_indexes)$
  $events \leftarrow get\_events(ID_{sink}, sink\_to\_events\_map)$
  for ID_{event} in $events$ **do**
    if $counter(ID_{event}, sinks\_to\_events\_counter) \leq max\_load$ **then**
      $activity \leftarrow get\_activity(ID_{event}, sink\_to\_events\_activity)$
      $add\_async\_circular\_buffer(ID_{event}, activity)$
    end if
  end for
end while
